# Impact of iron supplementation on patient outcomes for women with abnormal uterine bleeding: a protocol for a systematic review and meta-analysis

**DOI:** 10.1186/s13643-023-02222-4

**Published:** 2023-07-14

**Authors:** Hasmik Nazaryan, Megan Watson, Dana Ellingham, Swarni Thakar, Andrea Wang, Menaka Pai, Yang Liu, Bram Rochwerg, Nadia Gabarin, Donald Arnold, Emily Sirotich, Michelle P. Zeller

**Affiliations:** 1 Michael G. DeGroote Centre for Transfusion Research, McMaster University, 1200 Main Street West HSC-3H50, Hamilton, ON L8N 3Z5 Canada; 2 Department of Medicine and Department of Health Research Methods, Evidence and Impact, McMaster University, Hamilton, ON Canada; 3 Department of Medicine, McMaster University, Hamilton, ON Canada; 4 Canadian Blood Services, Ottawa, Canada

**Keywords:** Systematic review, Meta-analysis, Iron supplementation, Abnormal uterine bleeding, Heavy menstrual bleeding, Menorrhagia, Iron deficiency, Iron deficiency anemia, Red blood cell transfusion

## Abstract

**Background:**

Abnormal uterine bleeding (AUB), which includes heavy menstrual bleeding (HMB), is a common condition placing women at increased risk for developing iron deficiency and iron deficiency anemia (IDA). Depletion of iron stores has negative implications on physical, social, and emotional health, as well as quality of life. Iron supplements are safe, effective, and readily available, while red blood cell (RBC) transfusions have inherent risks including infectious and immune reactions. Despite high prevalence of IDA among women with AUB, there are limited studies on the impact of iron therapies on patient outcomes. This systematic review and meta-analysis will evaluate the impact of iron supplementation on patient outcomes for women with AUB, when compared to combination therapy, no intervention, placebo, or standard of care.

**Methods:**

We will conduct a systematic review and meta-analysis of randomized controlled trials and observational studies evaluating the impact of iron interventions on patient outcomes for women with AUB. Systematic literature searches will be conducted in major databases including MEDLINE, EMBASE, CENTRAL, CINAHL, and Web of Science. Studies assessing the impact of iron interventions on patient outcomes in women experiencing AUB, in comparison to combination therapy, no intervention, placebo, or standard of care, will be included in the review. Independent reviewers will screen for eligibility, assess risk of bias, and abstract data. Overall certainty of evidence for each outcome will be assessed using the GRADE approach. We will meta-analyze outcomes which are sufficiently homogeneous to summarize intervention effects and narratively synthesize nonhomogeneous outcomes. The main outcomes of interest are hemoglobin levels immediately prior to surgery and post-operatively, number of RBC transfusions, and adverse effects. Secondary outcomes will include length of hospital stay, intraoperative blood loss, adverse and side effects, quality of life, and iron indices.

**Discussion:**

This review will evaluate the impact of iron interventions on patient outcomes in women with IDA secondary to AUB with focus on changes in hematological and iron indices, red blood cell utilization, quality of life, cost of treatment, and adverse events. The results will inform evidence-based clinical practice for the management of iron deficiency and IDA secondary to AUB.

**Systematic review registration:**

PROSPERO CRD42019137282.

**Supplementary Information:**

The online version contains supplementary material available at 10.1186/s13643-023-02222-4.

## Background

### Heavy menstrual bleeding and abnormal uterine bleeding

Abnormal uterine bleeding (AUB) is defined as bleeding from the uterine corpus that is abnormal in volume, regularity, and/or timing, and which has been present for the majority of the last 6 months [1, 2]. In the clinical setting, AUB most commonly presents in the form of heavy menstrual bleeding (HMB) [3].

HMB is defined by the American College of Obstetricians and Gynecologists as excessive menstrual blood loss interfering with a woman’s physical, social, emotional, and/or material quality of life (QoL) [4–6]. HMB affects an estimated one-third of women of reproductive age globally; however, its prevalence can vary greatly depending on factors such as age and geographical origin [7]. Prevalence studies in European and Middle Eastern populations have found that HMB occurs in 15.2–37.9 % of women [8–11].

### Iron deficiency and iron deficiency anemia in women

If left untreated, blood loss from AUB can result in iron deficiency (ID) and iron deficiency anemia (IDA). Iron is an essential macronutrient that is crucial for the proper functioning of biological processes, including oxygen transport, cellular and mitochondrial respiration, electron transfer reactions, gene regulation, cell growth, and differentiation [12]. A depletion of total body iron stores can result from decreased availability (e.g., low consumption in the diet), increased loss (e.g., blood loss during menstruation), decreased absorption (e.g., from gut inflammation), or increased physiologic need (e.g., pregnancy) [13]. ID is the most common micronutrient deficiency globally, affecting nearly 2 billion people worldwide [13]. Women and children, especially in lower- and middle-income countries, are highly susceptible to ID where >20% of women experience ID during their reproductive years and are at a greater risk of developing IDA [14].

AUB is an important risk factor and principal cause of ID and IDA among women of reproductive age from high-income countries [15–18]. One study found that an estimated 63% of women with a form of AUB suffered from ID or IDA [8], whereas another determined that 60% of patients with AUB were severely iron deficient and 27% suffered from IDA [19]. In addition to iron repletion, management of AUB requires investigation into the underlying cause and may include various treatment options to reduce blood loss.

ID impacts neurocognitive, immunologic, and hematologic function [20]. Once ID progresses to IDA, there can be significant negative impacts on health, social, and economic well-being. Common sequelae of IDA include fatigue, weakness, decreased productivity [13], increased maternal morbidity and mortality, and neonatal and childhood complications, including preterm birth, low birth weight, and neonatal mortality [14].

### Iron repletion therapies as clinical management

Iron is present in iron-fortified foods (certain cereals), meat, chicken, and green leafy vegetables; however, diet alone is often insufficient for repletion and maintenance of adequate iron stores [16]. Iron repletion therapies are an important means of treatment for IDA resulting from AUB. The type and route of iron repletion depends on several factors including the severity, cause, comorbidities, and the preferences of the patient, as well as the availability of resources and the urgency of treatment. Oral iron supplementation and parenteral iron are the two main routes of iron repletion. Oral iron is an inexpensive and a widely available method; however, it can be poorly tolerated due to gastrointestinal side effects and/or may not replete and maintain iron stores. Intravenous (IV) iron is both safe and effective and can be used to administer a large amount of iron over the course of a short period of time with minimal risk of adverse reaction [14, 21, 22].

Red blood cell (RBC) transfusions are a limited resource with potential risk; while RBC tranfusions contain iron, they should be used exclusively to treat patients with IDA who are hemodynamically unstable or in whom oral and/or IV iron are contraindicated. Adverse reactions to RBC transfusion are of heightened concern in women of childbearing age because of the risk of alloimmunization and potential implications to a fetus in subsequent pregnancies [23]. Evidence supports the use of RBC transfusions as a last resort and not as a preferred method of treatment for IDA. Choosing Wisely Canada emphasizes the importance of avoiding RBC transfusions for ID when patients are hemodynamically stable and using iron supplementation [24, 25]. Previous studies have demonstrated inappropriate use of RBC transfusions to treat women with IDA [26, 27]. A Canadian retrospective chart review of 49 women with IDA who visited the emergency department identified that 42% of transfused RBC units were inappropriate for the indication and only 4% of patients received IV iron [26]. A retrospective cohort study of adolescent women with IDA secondary to HMB in the United States identified that 43% of patients received transfusions and only 7% were given IV iron [27]. More evidence to support the safe and effective use of iron interventions in women with IDA secondary to AUB is necessary to avoid inappropriate use of RBC transfusion.

### Objective

This review will evaluate the impact of iron supplementation compared to combination therapy, no iron treatment, placebo, or standard of care on patient outcomes in women with AUB. The results will inform evidence-based clinical practice for the management of ID and IDA secondary to AUB.

### Research question

Does the use of iron supplementation impact patient outcomes, primarily the change in hematological indices and number of RBC transfusions, in women experiencing AUB when compared to no treatment, placebo, combination therapy, or standard of care?

## Methods

The protocol has been registered in the PROSPERO database and assigned an identifier: CRD42019137282. This protocol was developed following the Preferred Reporting Items for Systematic Review and Meta-Analysis Protocols (PRISMA-P) statement checklist (see Additional file 1) [28].

### Data sources

Searches will be conducted in five databases for the completion of this systematic review: MEDLINE (Ovid), EMBASE (Ovid), Cochrane Central Register of Controlled Trials (CENTRAL), CINAHL, and Web of Science. A comprehensive list of terms related to iron and AUB will be developed to form search strategies specific to each database (see Additional file 2). Searches in all databases will be conducted on the same day and all identified studies will be included in title and abstract screening. There will be no restrictions based on language or publication status. Google Scholar will be searched using keywords. Ongoing trials will be identified using the World Health Organization (WHO) International Clinical Trials Registry Platform and ClinicalTrials.gov. For other unpublished articles, the ProQuest Dissertations and Theses databases and major conference proceedings in the fields of transfusion medicine, hematology, and surgery dating back 5 years will be searched using PapersFirst. These meetings include the International Society of Blood Transfusion Conference, Canadian Society for Transfusion Medicine, American Society Hematology, World Congress of International Society of Hematology, and the Canadian Hematology Society Meeting. OpenGrey will be used to search for grey literature. The reference lists of review articles identified through the literature search will be screened manually for relevant studies. Reference lists of included studies will also be screened manually for relevant studies.

### Eligibility criteria

Studies will be included in the review if they meet the following criteria. There will be no restrictions based on publication status or language. Studies published prior to the date of the final search will be considered for inclusion.

#### Study type

Randomized controlled trials (RCTs) and observational studies (retrospective or prospective) will be considered.

#### Participants

Studies including patients of reproductive potential age (in-patient or out-patient) with AUB, measured either objectively or subjectively, regardless of diagnoses, and co-morbidities, will be considered.

#### Intervention

Studies which assessed iron supplementation interventions in women with AUB will be reviewed for inclusion. Iron supplementation interventions are defined as iron in any compound (iron dextrose, iron sucrose, etc.) administered by any route (oral, IV, intramuscular, etc.) for all durations and with any co-interventions (erythropoietin stimulating agents, etc.). All doses of iron and iron administration schedules will be assessed.

#### Comparators

Studies that compare all routes of iron administration and iron delivered in combination with other compounds against each other, no iron treatment, placebo, or standard of care (as defined by the study) will be considered.

#### Outcome measures

The primary outcomes for this review are changes in hematological and iron indices, primarily hemoglobin and serum ferritin levels pre- and post-iron intervention. Secondary outcomes include number of RBC transfusions, iron indices, length of hospital stay, QoL, cost of treatment, and adverse events, where available.

### Exclusion criteria

Studies will be excluded if they are not in live human participants; if participants did not experience AUB; if participants were pregnant and/or underwent obstetrical surgery; if the study did not compare an iron intervention to placebo, no intervention, an alternate form of iron delivery, or iron in addition to another compound perioperatively; if iron was given for any reason other than increasing hemoglobin levels; or if the study is a case report or case series of <20 participants or a review article (not a primary study).

### Study selection

Two independent reviewers (H.N. and M.W.) will screen all titles and abstracts identified from the search and exclude studies based on the predefined eligibility criteria. Potentially relevant studies will then undergo a full-text review to determine final eligibility. Disagreements regarding inclusion will be resolved through discussion and with a third reviewer (E.S.), when necessary. Review authors will not be blinded to author or journal details during study selection. A PRISMA flowchart will be used to document the screening process and the reasons for exclusion [29].

### Data abstraction

Two data abstraction forms will be used to abstract data, one for included randomized control trials and another for included observational studies. These forms will be piloted using a representative sample of studies to determine ease of use, clarity, and appropriateness of form items. The most important results will be organized into a table of study characteristics. Two review authors (H.N. and M.W.) will independently abstract data and disagreements will be resolved through discussion and with a third reviewer (E.S.), when needed. Review authors will not be blinded to author or journal details during data abstraction.

Data to be abstracted includes study citation and author contact details, study design, duration and setting, sequence generation, allocation sequence concealment, blinding, other concerns about bias, number of participants, the diagnostic criteria, age, sex, and country of participants, the intervention details and number of intervention groups, number of participants allocated to each intervention group, outcomes at each time point collected and reported, outcome definitions, units of measurement, tool of measurement, and interpretation of scales if used as a tool, results of each outcome, missing data, estimate of effects with confidence interval and *p*-value, and subgroup analyses, funding sources, and references to any other relevant studies. Where there is missing data, reviewers will attempt to contact the study authors.

### Risk of bias assessment

The quality of included primary studies will be independently assessed by two reviewers (H.N and M.W.) using the Cochrane Risk of Bias assessment tool [30] for randomized trials and the Newcastle–Ottawa Scale for Risk of Bias Assessment for any included observational studies [31].

The Cochrane tool will be used to judge risk of bias in 7 specific domains and studies will be placed into one of three categories, “low,” “high,” and “unclear”. The 7 domains are selection bias due to inadequate random sequence generation or allocation concealment (2 domains per study), performance bias due to inadequate blinding of participants and personnel (to be assessed for each critical outcome per study), detection bias due to inadequate blinding of outcome assessment (to be assessed for each critical outcome per study), attrition bias due to incomplete outcome data (to be assessed for each critical outcome per study), reporting bias due to selective reporting (1 domain per study), and other bias not covered elsewhere (1 domain per study).

The Newcastle–Ottawa scale will be used to assess studies through a “star system” in three broad domains: the selection of the study groups, the comparability of the groups, and the ascertainment of either the exposure or outcome of interest for case–control or cohort studies respectively. Studies will be scored based on the number of stars that are tallied, with a maximum of 7 stars. Bias will be considered to be “serious” if less than 7, but greater than 4 stars. Bias will be considered to be “very serious” if less than or equal to 4 stars. A sensitivity analysis will be conducted excluding high ROB studies.

### Data analysis

A primary analysis will be conducted to investigate hemoglobin levels/anemia pre-operatively and post-operatively, number of RBC transfusions, and adverse events where possible. Aggregate level data will be used for this study. A quantitative synthesis, specifically a meta-analysis, will be used for outcomes which are sufficiently homogeneous to summarize intervention effects. Otherwise, a narrative synthesis of study findings will be reported for outcomes which are not sufficiently homogeneous. In addition, the population characteristics, type of interventions, and type of outcomes will be narratively described. Results may be adjusted for timing of interventions and duration. We anticipate that performing a meta-analysis will not be possible for all outcomes measured in this review.

Where a quantitative synthesis is possible, results will be pooled using a random-effects meta-analysis to account for variation in effect size amongst studies, with standardized mean differences for continuous outcomes and risk ratios for categorical outcomes. Pooled hazard ratios (HR) with 95% confidence intervals (CIs) and two-sided *p*-values will be calculated for each meta-analyzed outcome. All final results of meta-analyses will be summarized using forest plots. If a meta-analysis is performed, heterogeneity among included systematic reviews will be quantified using inconsistency index (*I*^2^) and *p*-values from the chi-square test for homogeneity. All analyses will be performed using statistical computing software R-342.

A secondary analysis will be conducted to investigate length of hospital stay, intraoperative blood loss, adverse and side effects, quality of life, and iron indices where feasible. Subgroup analyses will also be conducted to analyze patient outcomes according to dosage of iron supplementation, type of iron supplementation, route of iron supplementation, timing of intervention, other forms of red blood cell mass optimization or hemostasis optimization, age, and geographic region, if feasible. Additional post-hoc analyses may be indicated.

Results of this review will be reported according to the Preferred Reporting Items for Systematic Reviews and Meta-Analyses (PRISMA) guidelines [28]. The reasons for any protocol amendments will be documented in the full review.

### GRADE certainty assessment

Overall certainty of evidence for each outcome of interest, where meta-analyzable, will be assessed using the Grading of Recommendations Assessment, Development and Evaluation (GRADE) system. The GRADE system classifies the certainty of the pooled estimate of effect as high, moderate, low or very low through evaluation of the following criteria: individual study risk of bias, directness, consistency, precision and publication bias. An evidence profile will be produced to summarize the results using the GRADEpro software [32].

## Discussion

Women suffering from AUB, including HMB, are at an increased risk of developing ID and IDA, conditions which have negative physical and emotional consequences. Diet alone is often insufficient for the repletion of iron stores; therefore, iron supplementation is a crucial aspect of treating IDA secondary to AUB. RBC transfusions carry unnecessary risks and should be avoided when alternate treatment options are available. The restoration of hematological parameters in women with AUB has been found to have significant implications for general health and well-being by improving QoL. Despite its high prevalence among women of reproductive age, there are limited studies on the impact of iron treatment for the management of ID and IDA secondary to AUB.

This systematic review will synthesize evidence from available RCTs and observational studies on the impact of iron interventions on patient outcomes, particularly hematological, and iron indices and the use of RBC transfusion in women experiencing AUB. This review will identify which iron interventions, if any, might be effective in improving patient outcomes compared to combination therapy, no iron treatment, placebo, or standard of care. The results will inform evidence-based treatment decisions for patients with ID/IDA as a result of AUB. Findings from this study could be used to inform clinicians and patients, as well as clinical protocols regarding the use of iron interventions for the management of ID/IDA in women with AUB.

Possible limitations of this systematic review include the potential for high heterogeneity among included studies, namely in terms of the different iron interventions used. This type of heterogeneity may limit the validity of the results and the scope of the analysis. A disproportionately higher number of studies available in North America in comparison to other regions of the world may limit generalizability. Another limitation may be low availability of studies that compare iron interventions to placebo, due to ethical concerns. It may also be difficult to find studies with iron administered as a single comparator since it is often given in combination with other therapies, thus confounding the results.

While language will not be an exclusion criterion in this search strategy to minimize the risk of language bias, translations may not be available. The robust methodological process of the systematic review and the use of GRADE to evaluate the certainty of evidence supporting treatment effects will contribute to the strength of the review.

## Supplementary Information

**Additional file 1.** PRISMA-P Checklist. This checklist addresses the recommended items to report in a systematic review protocol and references their locations within the text.

**Additional file 2.** MEDLINE (Ovid) Search Strategy. The search strategy developed for MEDLINE (Ovid) will be modified for use in other databases.

## Data Availability

Not applicable.

## Abbreviations

AUB: Abnormal uterine bleeding
CENTRAL: Cochrane Central Register of Controlled Trials
CI: Confidence interval
FIGO: Fédération International de Gynécologie et d'Obstétrique
GRADE: Grading of Recommendations Assessment, Development and Evaluation
HMB: Heavy menstrual bleeding
HR: Hazard ratio
ID: Iron deficiency
IDA: Iron deficiency anemia
IV: Intravenous
PRISMA: Preferred Reporting Items for Systematic Review and Meta-Analysis
PRISMA-P: Preferred Reporting Items for Systematic Review and Meta-Analysis Protocols
QoL: Quality of life
RBC: Red blood cell
RCT: Randomized controlled trial
WHO: World Health Organization
