# Supplementary material for: Impact of iron supplementation on patient outcomes for women with abnormal uterine bleeding: a protocol for a systematic review and meta-analysis
Source: Syst Rev. 2023 Jul 14;12:121. doi: 10.1186/s13643-023-02222-4 (PMC10347734; doi:10.1186/s13643-023-02222-4)
Supplement: Supplementary file 2 — Additional file 2. MEDLINE (Ovid) Search Strategy. The search strategy developed for MEDLINE (Ovid) will be modified for use in other databases. [file 13643_2023_2222_MOESM2_ESM.docx]

**Additional File 2:** MEDLINE (Ovid) Search Strategy

1. Menorrhagia/ or Menstruation Disturbances/ or Hypermenorrhea/

2. menorrhag*.mp. or hypermenorrh*.mp. or Menometrorrhag*.mp. or Metromenorrhag*.mp. or Metrorrhag*.mp.

3. (menstrua* adj3 (bleed* or disorder or blood*)).mp.

4. (heavy adj2 (bleed* or period or menses or menstrua* or uterus or uterine)).mp.

5. (abnormal adj2 (bleed* or period or menses or menstrua* or uterus or uterine)).mp.

6. (dysfunctional adj2 (bleed* or period or menses or menstrua* or uterus or uterine)).mp.

7. (excessive adj2 (bleed* or period or menses or menstrua* or uterus or uterine)).mp.

8. (irregular adj2 (bleed* or period or menses or menstrua* or uterus or uterine)).mp.

9. Iron/ or exp Iron Compounds/ or iron.mp. or ferric*.mp. or ferrous*.mp.

10. (1 OR 2 OR 3 OR 4 OR 5 OR 6 OR 7 OR 8) AND 9
